# Exploring the predictive value of lesion topology on motor function outcomes in a porcine ischemic stroke model

**DOI:** 10.1038/s41598-021-83432-5

**Published:** 2021-02-15

**Authors:** Kelly M. Scheulin, Brian J. Jurgielewicz, Samantha E. Spellicy, Elizabeth S. Waters, Emily W. Baker, Holly A. Kinder, Gregory A. Simchick, Sydney E. Sneed, Janet A. Grimes, Qun Zhao, Steven L. Stice, Franklin D. West

**Affiliations:** 1 Regenerative Bioscience Center, University of Georgia, Athens, GA USA; 2 Department of Animal and Dairy Sciences, University of Georgia, Athens, GA USA; 3 Biomedical and Health Sciences Institute, Neuroscience Program, University of Georgia, Athens, GA USA; 4 Department of Physics, University of Georgia, Athens, GA USA; 5 Aruna Bio Inc, Athens, GA USA; 6 Department of Small Animal Medicine and Surgery, College of Veterinary Medicine, University of Georgia, Athens, GA USA

**Keywords:** Magnetic resonance imaging, Imaging, Neuroscience, Motor control, Stroke

## Abstract

Harnessing the maximum diagnostic potential of magnetic resonance imaging (MRI) by including stroke lesion location in relation to specific structures that are associated with particular functions will likely increase the potential to predict functional deficit type, severity, and recovery in stroke patients. This exploratory study aims to identify key structures lesioned by a middle cerebral artery occlusion (MCAO) that impact stroke recovery and to strengthen the predictive capacity of neuroimaging techniques that characterize stroke outcomes in a translational porcine model. Clinically relevant MRI measures showed significant lesion volumes, midline shifts, and decreased white matter integrity post-MCAO. Using a pig brain atlas, damaged brain structures included the insular cortex, somatosensory cortices, temporal gyri, claustrum, and visual cortices, among others. MCAO resulted in severely impaired spatiotemporal gait parameters, decreased voluntary movement in open field testing, and higher modified Rankin Scale scores at acute timepoints. Pearson correlation analyses at acute timepoints between standard MRI metrics (e.g., lesion volume) and functional outcomes displayed moderate R values to functional gait outcomes. Moreover, Pearson correlation analyses showed higher R values between functional gait deficits and increased lesioning of structures associated with motor function, such as the putamen, globus pallidus, and primary somatosensory cortex. This correlation analysis approach helped identify neuroanatomical structures predictive of stroke outcomes and may lead to the translation of this topological analysis approach from preclinical stroke assessment to a clinical biomarker.

## Introduction

Middle cerebral artery occlusion (MCAO) is the most common cause of ischemic stroke and one of the leading causes of mortality and long-term disability worldwide^1^. Nearly half of survivors with large territory middle cerebral artery (MCA) strokes have permanent deficits and fail to regain functional independence^2^. Magnetic resonance imaging (MRI) is commonly used in clinical practice to evaluate stroke pathology, determine injury severity, and predict patient outcomes. However, standard MRI measures, such as lesion volume and midline shift, alone have limited prognostic value with respect to functional deficit type, severity, and recovery^3–8^. Including stroke lesion location in relation to specific structures that coordinate with functional tasks (e.g., a lesioned primary somatosensory cortex leads to loss of limb coordination in walking) is likely to increase the predictive power of acute MRI metrics as biomarkers of stroke outcomes^9^.

The prognostic value of lesion volume, the most commonly used biomarker to assess stroke severity and predict patient outcomes, is only moderate for motor impairments^5^. The correlation between lesion volume and quality of life measurements, such as modified Rankin Scale (mRS), have also proven to be limited^3,4^. To ultimately strengthen the prognostic value of MRI, lesion location has been assessed using topographic maps and resulted in increased predictive power^6,10–18^. Using a voxel-based lesion mapping technique, infarcts in the corona radiata, internal capsule, and insula were associated with worse mRS scores in patients with an MCAO ischemic stroke^6^. Lesions in the insular ribbon, lentiform nuclei, and middle corona radiata resulted in poor recovery based on the National Institutes of Health Stroke Scale (NIHSS) scoring^18^. Additionally, regionalized infarction in the left basal ganglia and frontal lobe were negatively correlated with functional independence scores^1^. Overall, these findings indicate that lesion location is a key metric to determine an accurate prognosis of functional recovery post-stroke. Since discrepancies regarding lesion size and location in clinical stroke recovery exist as well as a void in viable translational models, the current study aims to characterize structures lesioned by an MCAO and evaluate their impact on stroke outcomes.

The Stroke Therapy Academic Industry Roundtable (STAIR) recommends using permanent occlusion gyrencephalic preclinical models, such as a porcine permanent MCAO model, and considering clinically relevant biomarkers, such as MRI detected lesion volume and location, to assess new treatments^19,20^. The pig model serves as a valuable large animal system to study the effects of MCAO on specific structures due to the comparable brain size and similar cerebral composition (e.g., gray to white matter ratio) between pigs and humans^21,22^, and the ability to utilize a standardized porcine MRI brain atlas^23^. Critical brain structures present in the human and pig brain such as the hippocampus, caudate, and putamen are found in different locations, orientations, or absent in the rodent brain, the most commonly used stroke animal model^21,24–26^. For example, in the human and pig, the striatum is separated into two individual structures, the caudate nucleus and putamen, while the caudate nucleus and putamen are indistinguishable from each other in the rodent^27,28^. These unique differences in critical structures in the mouse brain relative to the human and pig brain are likely to have a profound effect on stroke outcomes. Our research group has developed a more translational porcine MCAO model that displays stroke pathophysiology comparable to humans including lesioning, cytotoxic and vasogenic edema, and white matter damage as determined by MRI^29–33^. In addition, we have demonstrated that the porcine MCAO model displays behavioral and motor function deficits similar to human patients^34–37^. Building upon the unique strengths of the porcine MCAO model and initial advancements utilizing basic acute MRI measures to predict functional outcomes, we assessed the potential of lesion topology as an important MRI variable to advance functional prognostication.

In this exploratory study, we utilized MRI to evaluate acute clinical pathologies, including lesion volume and midline shift, and lesioning of individual brain structures to determine their potential in predicting chronic motor function outcomes in a porcine model of ischemic stroke. These findings suggest that including regional damage information in acute clinical evaluations of stroke patients is beneficial in determining acute motor deficits.

## Results

### MCAO resulted in lesioning, hemispheric swelling/atrophy, and decreased white matter integrity

**Figure 1:**
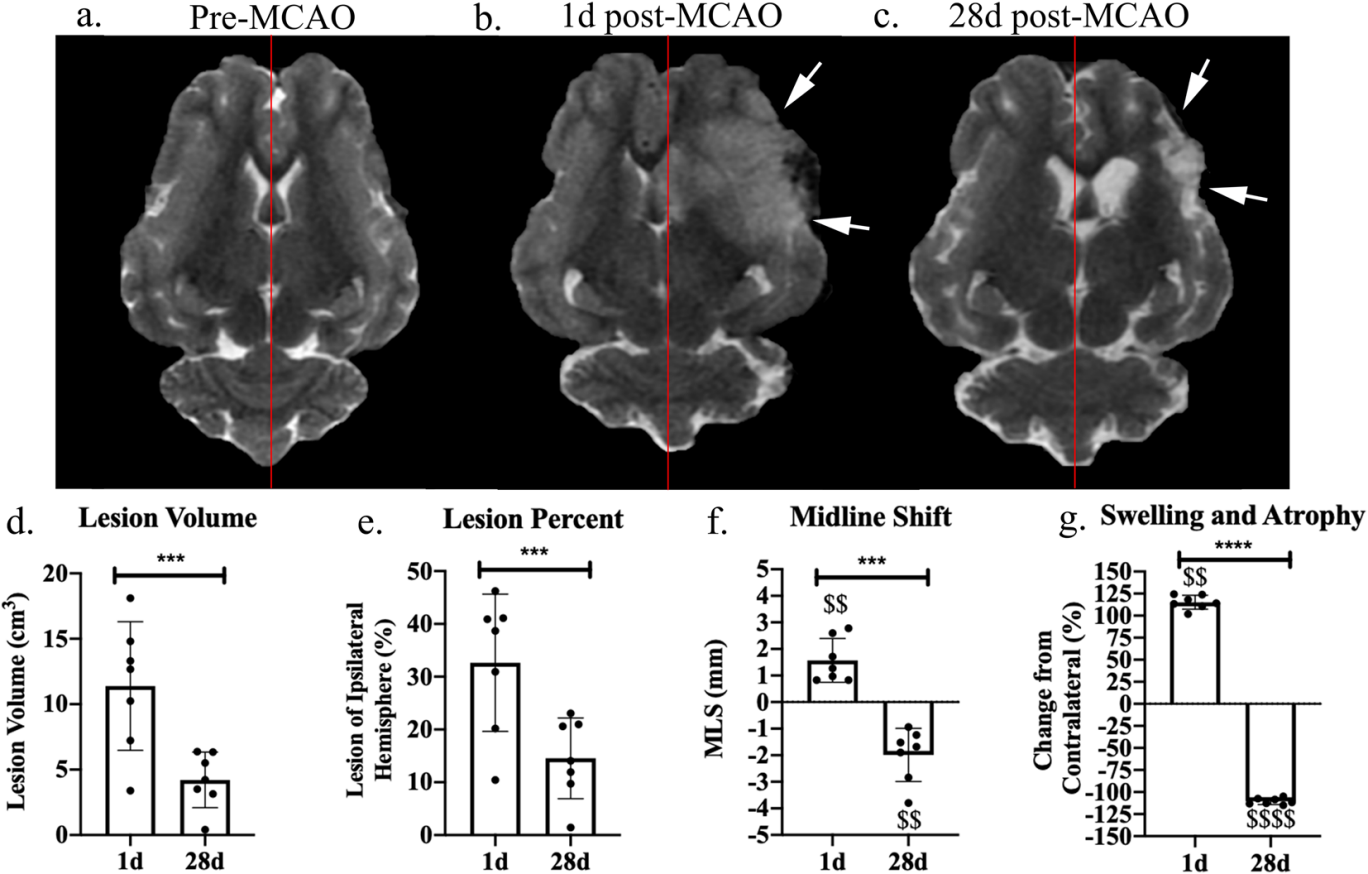
Canonical MRI measurements of MCAO induced ischemic stroke at 1d and 28d post-MCAO. T2W images prior to MCAO reveal homology between the hemispheres (**a**). At 1d (**b**) and 28d (**c**) post-MCAO, T2W images exhibited territorial hyperintense lesions indicated by white arrows and a shift from the natural midline (red lines indicate natural midline). Lesion volume (**d**), lesion volume as a percent of the ipsilateral hemisphere (**e**), midline shift (MLS) (**f**), and hemispheric changes (**g**) were quantified at 1d and 28d post-MCAO. *** and **** indicated statistically different between timepoints. $$ and $$$$ indicated statistically different from hypothetical normal.

To assess the dynamic pathophysiological response of an MCAO ischemic stroke, T2Weighted (T2W) MRI sequences were evaluated. Visual assessments of pre-MCAO pig brains showed no observable damage (Fig. 1a). At 1 day (d) post-MCAO, the MCAO group displayed substantial lesioning (hyperintense region, white arrows), a midline shift towards the unaffected contralateral hemisphere, and decreased ventricle size due to cerebral swelling (Fig. 1b). By 28d post-MCAO, animals showed a decrease in lesion size, a midline shift towards the ipsilateral hemisphere, and increased ventricle size due to tissue atrophy (Fig. 1c). Quantitative lesion volume analysis showed significant brain lesioning 1d post-MCAO with an average volume of 11.39 ± 4.91 cm^3^ and by 28d post-stroke, lesion volume size (4.22 ± 2.12 cm^3^) was significantly (*p* = 0.0010) reduced (Fig. 1d). To account for potential animal variability, lesion volume was evaluated as a percent of the ipsilateral hemisphere. At 1d post-MCAO, the lesion occupied 32.64 ± 12.99% of the ipsilateral hemisphere and by 28d post-MCAO, the lesion occupied 14.54 ± 7.66% (*p* = 0.0004) (Fig. 1e). In MCAO pigs, the ipsilateral hemisphere showed 1.57 ± 0.83 mm midline shift towards the contralateral hemisphere at 1d post-MCAO, indicating hemispheric swelling and was different from normal (*p* = 0.0024). At 28d post-MCAO, the ipsilateral hemisphere showed a midline shift of  − 1.99 ± 1.00 mm away from the contralateral hemisphere (1d vs 28d post-MCAO, *p* = 0.0003), indicating hemispheric atrophy and was different from normal (*p* = 0.0019) (Fig. 1f). At 1d post-MCAO, animals showed hemispheric swelling with a 15.17 ± 7.89% increase in ipsilateral hemisphere volume and was different from normal (*p* = 0.0022). Cerebral atrophy was observed at 28d post-MCAO with a 10.41 ± 3.63% decrease in ipsilateral hemisphere volume from 1d post-MCAO (*p* < 0.0001) (Fig. 1g) and was different from normal (*p* < 0.0001).

Axial diffusion tensor imaging (DTI) images in pre-MCAO animals depict major white matter tracts from a medial slice of the pig brain and showed an intact ipsilateral (right side, white arrow) and contralateral (left) CC with fractional anisotropy (FA) values of 0.42 ± 0.10 and 0.45 ± 0.13, respectively (*p* > 0.05) (Supplementary Fig. 2a, d). At 1d post-MCAO, the ipsilateral CC was disrupted and showed a significant (*p* = 0.0347) decrease in FA (0.33 ± 0.04) compared to the contralateral CC (0.47 ± 0.12) (Supplementary Fig. 2b, e). The ipsilateral CC had recovered FA by 28d post-MCAO with ipsilateral FA of 0.39 ± 0.10 and contralateral FA of 0.41 ± 0.05 (p > 0.05) (Supplementary Fig. 2c, f). The corpus callosum (CC) is responsible for interhemispheric communications and, due to the location of the ischemic lesion, we found the CC to be impacted. The structural integrity of the CC after stroke has been shown to be repeatedly associated with motor deficits^38,39^. Overall, standard MRI analyses showed significant lesioning along with hemispheric swelling, subsequent atrophy, and decreased white matter integrity post-MCAO.

**Figure 2:**
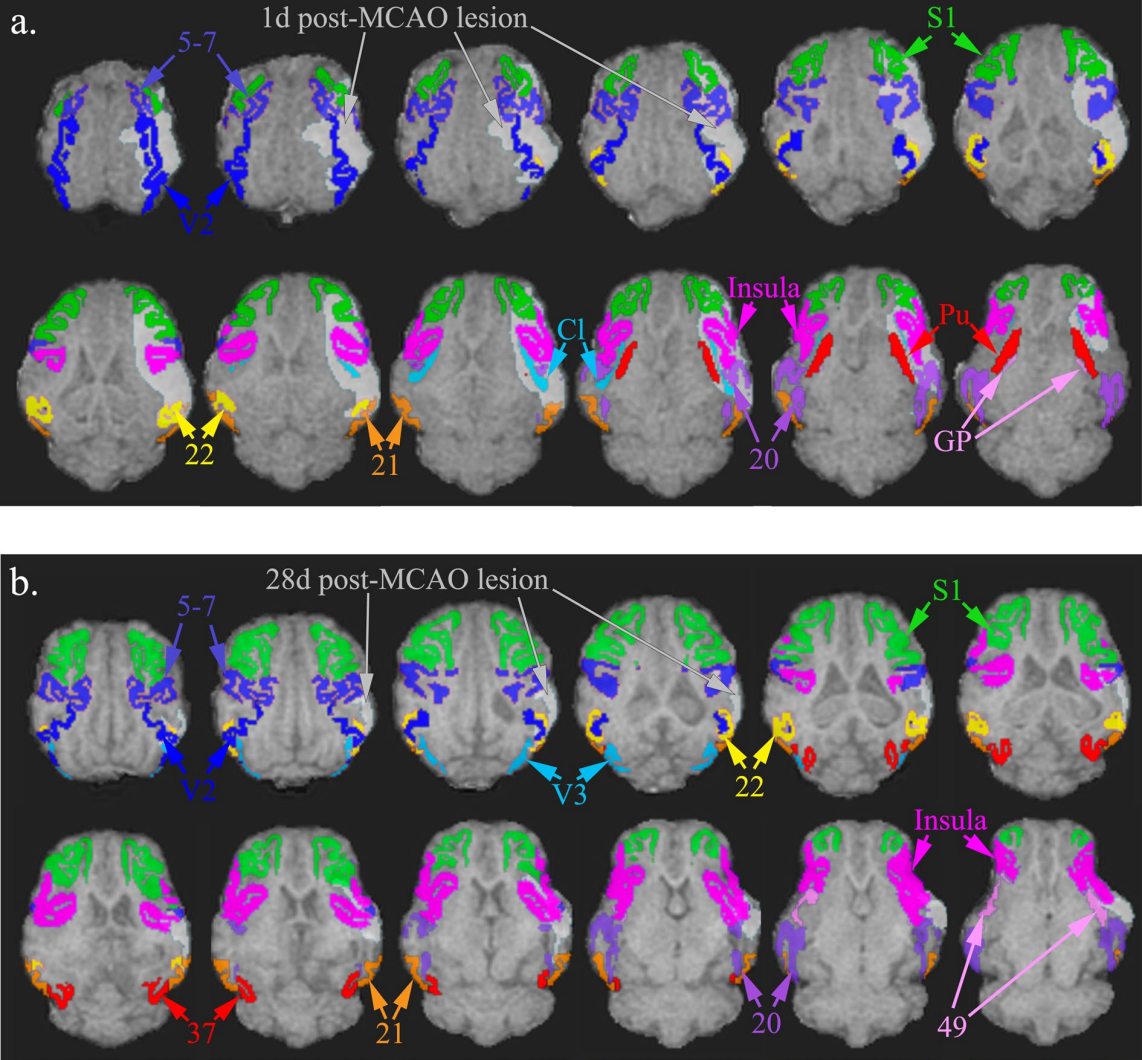
Structures most highly lesioned by MCAO in a porcine ischemic stroke model. Images depict axial T1 brain overlay with entire bilateral structures most affected by the lesion. The structures with the highest percentage of infarction (percent of structure, PoS) at 1d post-MCAO in descending order include the claustrum (*Cl*), inferior temporal gyrus (*Brodmann’s Area 20*), superior temporal gyrus (*Brodmann’s Area 22*), insular cortex (*Insula*), middle temporal gyrus (*Brodmann’s Area 21*), putamen (*Pu*), somatosensory association cortex (*Brodmann’s Area 5–7*), secondary visual cortex (*V2*), globus pallidus (*GP*), and primary somatosensory cortex (*S1*) (**a**). At 28d post-MCAO, the structures with the highest level of lesioning included the middle temporal gyrus (*Brodmann’s Area 21*), superior temporal gyrus (*Brodmann’s Area 22*), inferior temporal gyrus (*Brodmann’s Area 20*), somatosensory association cortex (*Brodmann’s Area 5–7*), insular cortex (*Insula*), associative visual cortex (*V3*), primary somatosensory cortex (*S1*), prepiriform area (*Brodmann’s Area 49*), fusiform gyrus (*Brodmann’s Area 37*), and secondary visual cortex (*V2*) (**b**).

### Ischemic lesioning spanned the thalamic nuclei, striatum, limbic system, and multiple Brodmann’s areas

The ischemic lesion affected multiple major brain regions including the thalamic nuclei, striatum, limbic system, and various Brodmann’s areas. A regional pixel analysis was completed to calculate the percentage of each structure lesioned. The structures with the highest percentage of infarction (percent of structure, PoS) at 1d post-MCAO included the claustrum (65.91 ± 29.68%), inferior temporal gyrus (62.15 ± 27.67%), superior temporal gyrus (58.91 ± 36.32%), insular cortex (54.45 ± 31.35%), middle temporal gyrus (44.43 ± 24.49%), putamen (44.00 ± 22.23%), somatosensory association cortex (43.05 ± 27.06%), secondary visual cortex (40.57 ± 28.62%), globus pallidus (34.52 ± 28.61%), and primary somatosensory cortex (33.48 ± 23.73%) (Fig. 2a). At 28d post-MCAO, the structures with the highest level of lesioning included the middle temporal gyrus (39.84 ± 31.80%), superior temporal gyrus (31.14 ± 17.55%), inferior temporal gyrus (18.19 ± 13.55%), somatosensory association cortex (16.78 ± 11.46%), insular cortex (15.95 ± 9.28%), associative visual cortex (10.02 ± 14.48%), primary somatosensory cortex (8.44 ± 8.92%), prepiriform area (7.84 ± 5.85%), fusiform gyrus (6.82 ± 10.44%), and secondary visual cortex (5.83 ± 6.30%) (Fig. 2b). The percent of the identified lesion (PoL) in each structure was quantified to represent the percent of the lesion that affected specific brain structures and characterized stroke location. At 1d post-MCAO, the structures that occupied the lesion included the insular cortex (8.45 ± 5.09%), primary somatosensory cortex (6.33 ± 4.82%), secondary visual cortex (6.02 ± 4.34%), inferior temporal gyrus (5.16 ± 4.65%), somatosensory association cortex (4.98 ± 2.87%), putamen (3.22 ± 1.58%), middle temporal gyrus (2.59 ± 2.97%), superior temporal gyrus (2.36 ± 1.68%), parahippocampal cortex (2.06 ± 3.00%), claustrum (1.94 ± 0.71%), prepiriform area (1.44 ± 0.61%), amygdala (1.23 ± 1.95%), and primary visual cortex (1.08 ± 1.36%) (Supplementary Fig. 3a). By 28d post-MCAO, PoL structures that occupied the lesion included the insular cortex (7.95 ± 6.02%), primary somatosensory cortex (5.64 ± 6.36%), somatosensory association cortex (5.41 ± 3.59%), middle temporal gyrus (4.23 ± 3.01%), inferior temporal gyrus (3.27 ± 2.72%), superior temporal gyrus (3.15 ± 1.87%), secondary visual cortex (2.07 ± 1.93%), parahippocampal cortex (1.42 ± 2.34%), and prepiriform area (1.30 ± 1.06%) (Supplementary Fig. 3b). A complete list of lesioned structures (PoS and PoL) (Mean ± standard deviation (SD)) are detailed in Supplementary Table 1. Notably, 100% of pigs had lesioning in the claustrum, putamen, insular cortex, secondary visual cortex, inferior temporal gyrus, superior temporal gyrus, parahippocampal cortex, and prepiriform area (Table 1).

**Table 1:** Percentage of pigs with lesion in each structure.

Region	Structure	Pigs effected at 1d post-MCAO (%)	Pigs effected at 28d post-MCAO (%)
Thalamus	Pulvinar nuclei	42.9	–
	Reticular thalamic nucleus	42.9	–
	Ventral anterior thalamic nucleus	14.3	–
	Ventral posterior thalamic nucleus	42.9	–
Striate system	Caudate nucleus	71.4	–
	Claustrum	100	28.6
	Globus pallidus	71.4	–
	Putamen	100	14.3
Limbic system	Fornix	85.7	28.6
	Hippocampus	85.7	14.3
	Subiculum	42.9	–
	Amygdala	71.4	28.6
Brodmann’s area	Primary somatosensory cortex	71.4	71.4
	Primary motor cortex	28.6	–
	Somatosensory association cortex	85.7	100
	Dorsolateral prefrontal cortex	42.9	14.3
	Anterior prefrontal cortex	28.6	–
	Orbitofrontal cortex	14.3	–
	Insular cortex	100	85.7
	Primary visual cortex	71.4	28.6
	Secondary visual cortex	100	85.7
	Associative visual cortex	85.7	57.1
	Inferior temporal gyrus	100	100
	Middle temporal gyrus	85.7	100
	Superior temporal gyrus	100	85.7
	Dorsal anterior cingulate cortex	14.3	–
	Anterior entorhinal cortex	85.7	14.3
	Parahippocampal cortex	100	71.4
	Fusiform gyrus	71.4	42.9
	Prepiriform area	100	100

Identified structures were separated into regions including the thalamus, striate system, limbic system, and Brodmann’s Areas. Activated pixels in each structure signified positive lesion. Lesion locations were identified at 1d and 28d post-MCAO. 100% signifies that all pigs (n = 7) had lesion in structure.

**Figure 3:**
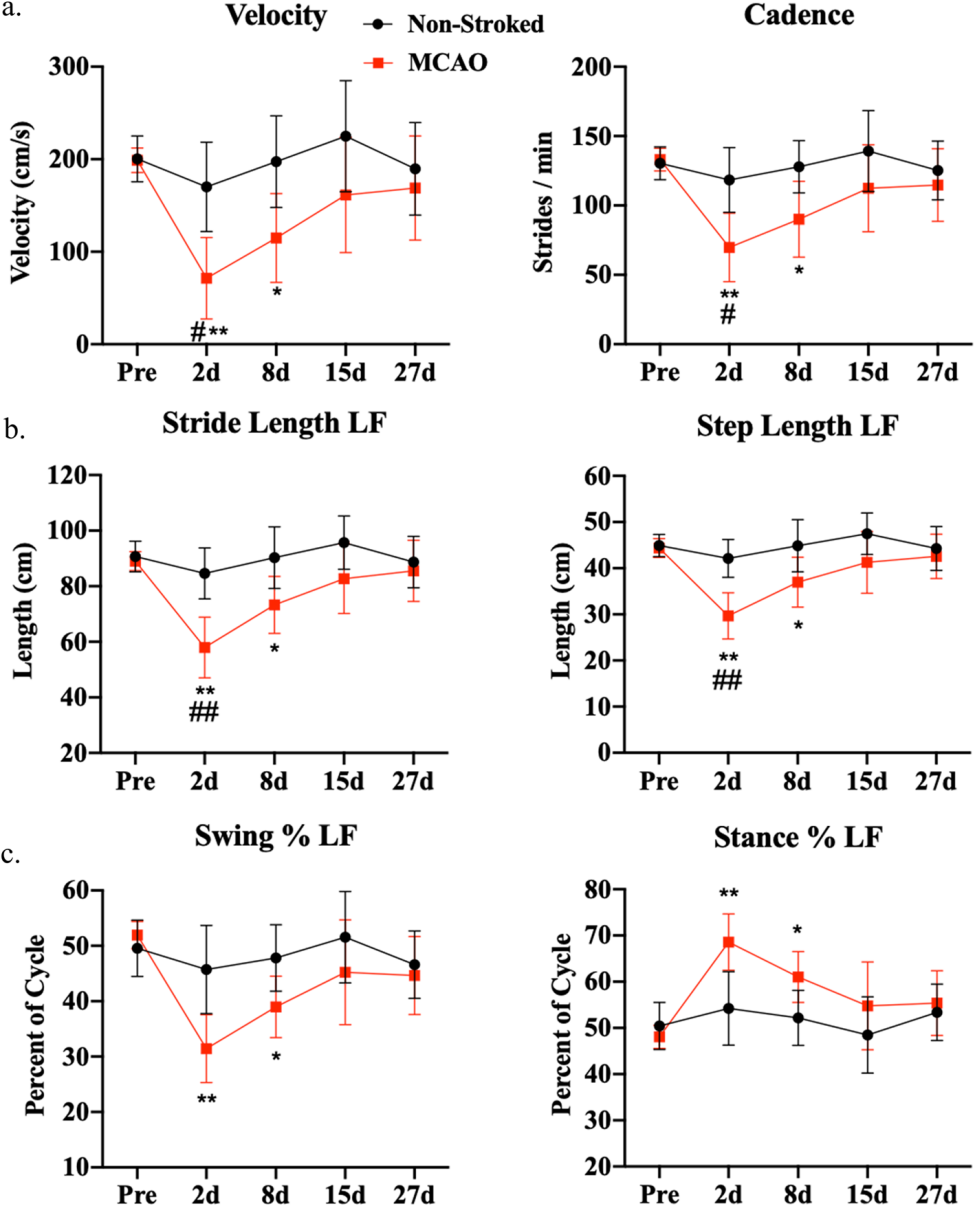
MCAO resulted in acute functional gait deficits. At 2d and 8d post-MCAO, MCAO pigs exhibited significant decreases in velocity (**a**), cadence (**a**), stride length (**b**), step length (**b**), swing percent (**c**), and an increase in stance percent (**c**) relative to pre-MCAO values. At 15d and 27d post-MCAO, there were no significant differences compared to pre-stroke, which was indicative of functional recovery (*p* > 0.05). At 2d post-MCAO, MCAO pigs exhibited significantly different velocity (**a**), cadence (**a**), stride length (**b**), and step length (**b**) relative to non-stroked controls. Non-stroked controls had no significant changes over the course of the study (*p* > 0.05). * and ** indicated statistical difference from pre-MCAO values. # and ## indicated statistically different between groups.

### MCAO induced functional gait deficits

To assess changes in motor function, gait analysis was performed on all animals pre-MCAO to establish a baseline and collected over a 27-day period following MCAO. Compiled functional outcome (gait and behavior) parameters definitions that were analyzed in this study can be found in Supplementary Table 2. The MCAO group showed significant decreases in the left front (LF) limb (contralateral to MCAO) at 2d post-MCAO in velocity (Fig. 3a; Pre: 198.78 ± 13.24 cm/s vs. Day 2: 71.49 ± 44.22 cm/s, *p* = 0.0048) and cadence (Fig. 3a; Pre: 133.01 ± 8.23 stride/min vs. Day 2: 69.78 ± 24.61 stride/min, *p* = 0.0081) as compared to pre-MCAO. In addition, there were significant decreases at 2d post-MCAO in the LF limb in stride length (Fig. 3b; Pre: 89.01 ± 3.42 cm vs. Day 2: 57.98 ± 10.96 cm, *p* = 0.0042), step length (Fig. 3b; Pre: 44.36 ± 2.05 cm vs. Day 2: 29.65 ± 5.00 cm, *p* = 0.0031), and swing percent (Fig. 3c; Pre: 51.93 ± 2.47% vs. Day 2: 31.45 ± 6.14%, *p* = 0.0034), along with an increase in stance percent (Fig. 3c; Pre: 48.06 ± 2.49% vs. Day 2: 68.55 ± 6.12%, *p* = 0.0034) relative to pre-MCAO. Velocity (Fig. 3a; Day 8: 114.77 ± 48.21 cm/s, *p* = 0.0168), cadence (Fig. 3a; Day 8: 90.03 ± 27.37 stride/min, p = 0.0247), stride length (Fig. 3b; Day 8: 73.29 ± 10.29 cm, *p* = 0.0289), step length (Fig. 3b; Day 8: 36.96 ± 5.39 cm, *p* = 0.0324), swing percent (Fig. 3c; Day 8: 38.98 ± 5.53%, *p* = 0.0104), and stance percent (Fig. 3c; Day 8: 61.03 ± 5.52%, *p* = 0.0104) continued to display deficits at 8d post-MCAO. No significant (*p* > 0.05) differences between the MCAO and non-stroked groups were measured pre-MCAO. Non-stroked pigs did not have any significant differences at any measured timepoints (Fig. 3). Stroked animals exhibited signs of recovery after 8d post-MCAO, showing no significant changes in measured parameters to non-stroked animals or pre-stroke values (*p* > 0.05). Gait parameters for the other three limbs, right front (RF), left hind (LH), and right hind (RH), are detailed in Supplementary Fig. 4.

**Figure 4:**
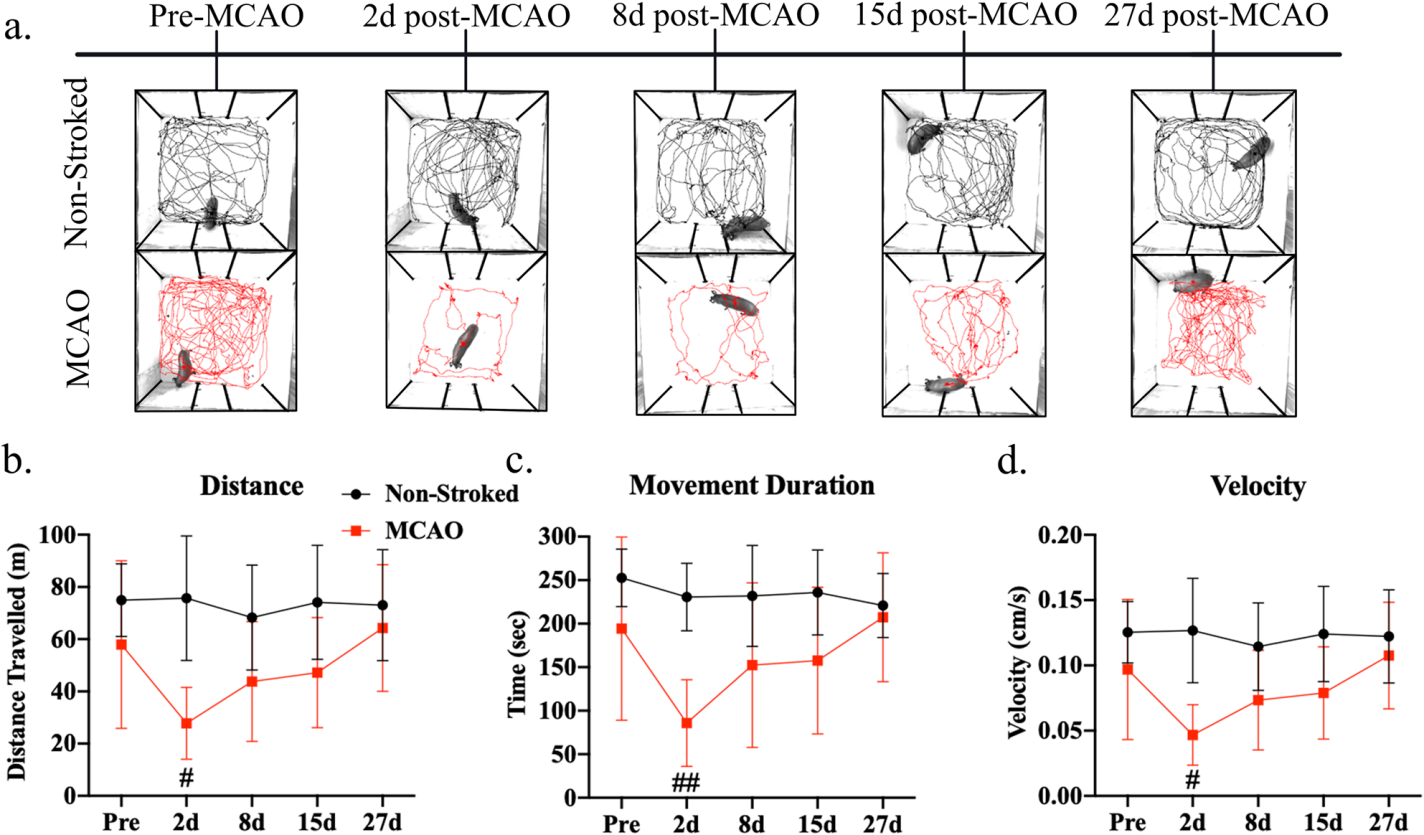
Stroke decreased acute voluntary movement in open field behavioral test. Representative 10-min movement tracings shown for non-stroked (black) and MCAO (red) pigs across all time points (**a**). At 2d post-MCAO, MCAO pigs had decreased distance traveled (**b**), movement duration (**c**), and average velocity (**d**) compared to non-stroked pigs. No significant differences were observed at 8d, 15d, and 27d post-MCAO in both groups (*p* > 0.05). # and ## indicated statistically different between groups.

### MCAO induced contralateral limb weakness

Changes in weight distribution in all four limbs due to stroke was evaluated by assessing deviations in total pressure index for each individual limb. Total pressure index is the sum of peak pressure values recorded from each activated sensor by a hoof during contact and is representative of weight distribution. The MCAO group showed a significant decrease in total pressure index at 2d post-MCAO in the LF limb (Supplementary Fig. 5a; Pre: 28.99 ± 1.66 vs. Day 2: 26.87 ± 2.07, *p* = 0.0224) with a corresponding increase in pressure in the RF limb (Supplementary Fig. 5b; Pre: 29.57 ± 1.95 vs. Day 2: 31.87 ± 2.30, *p* = 0.0413). By 8d post-MCAO, total pressure index showed no significant changes in measured parameters compared to pre-MCAO in the LF limb (Supplementary Fig. 5a; Pre: 28.99 ± 1.66 vs. Day 8: 27.96 ± 1.45, *p* = 0.1076) and the RF limb (Supplementary Fig. 5b; Pre: 29.57 ± 1.95 vs. Day 8: 29.65 ± 1.78, *p* = 0.9999). LH and RH limb total pressure indices did not change significantly compared to pre-MCAO (Supplementary Fig. 5c, d; *p* > 0.05). However, these results were expected in the pig model, as the forelimbs carried 60% of the body weight and, thus, are more susceptible to disruptions in weight distribution. Collectively, gait analysis provided quantifiable parameters showing functional gait deficits in weight distribution induced by MCAO.

**Figure 5:**
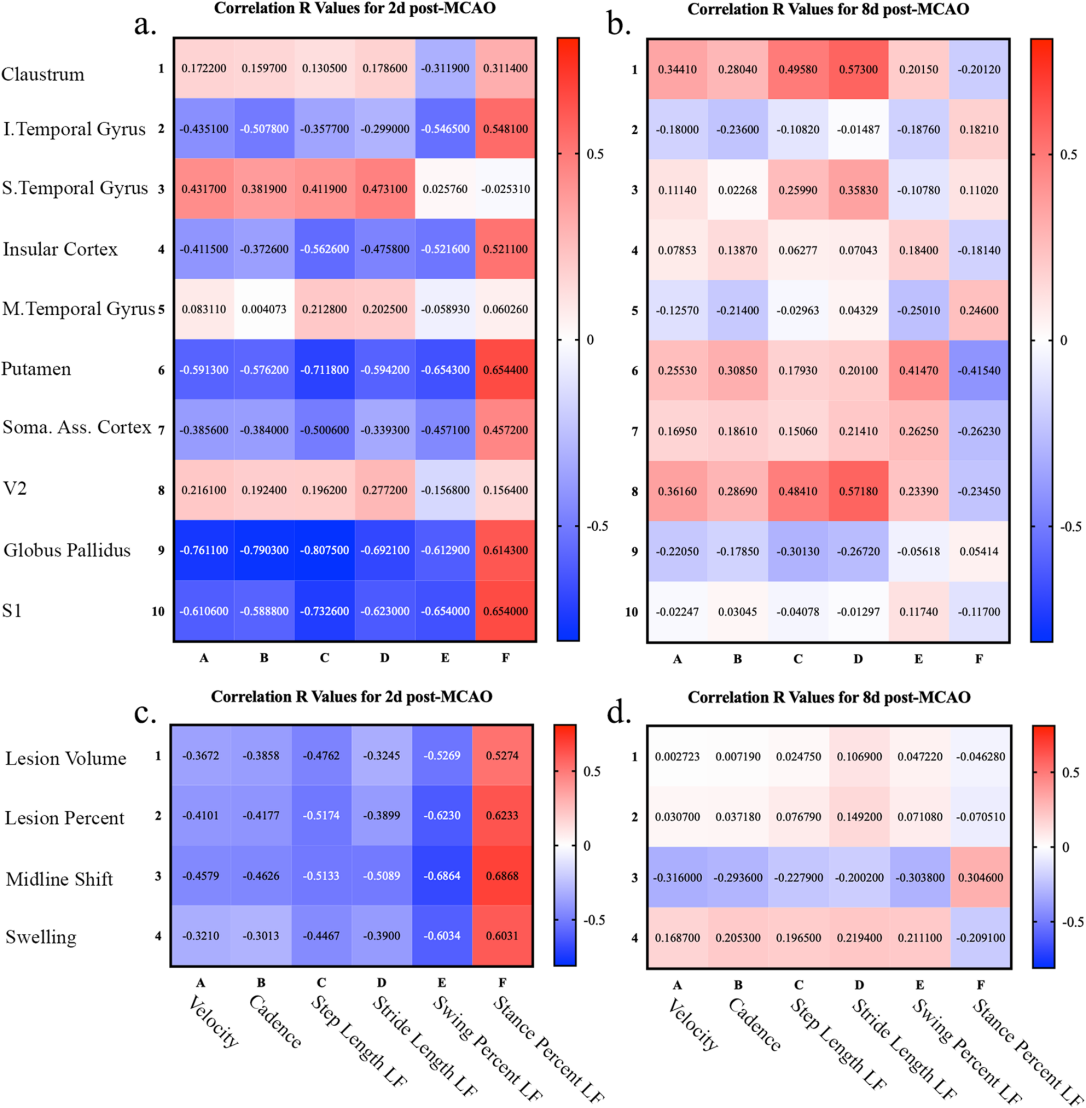
Location of stroke lesion in structures associated with motor coordination had prognostic value when evaluating motor functional outcomes. Pearson correlations between top PoS structures impacted by the lesion (y-axis) to gait functional outcomes (x-axis) were evaluated at 2d post-MCAO (**a**) and 8d post-MCAO (**b**). Pearson correlations between canonical MRI parameters (y-axis), including lesion volume, lesion percent of ipsilateral hemisphere, midline shift, and hemispheric swelling, and gait functional outcomes (x-axis) were evaluated at 2d post-MCAO (**c**) and 8d post-MCAO (**d**). R values were reported with cell color indicating correlation direction and strength. FDR p-values corrected for multiple comparisons can be found in Supplementary Table 3.

### MCAO pigs showed deficits in voluntary movement and behavioral scores

Open field testing was performed to assess behavioral changes post-stroke. Representative tracking images displayed decreased voluntary movement at 2d post-MCAO (Fig. 4a). At 2d post-MCAO, the MCAO group showed a significant decrease in distance traveled (Fig. 4b; Non-stroked: 75.70 ± 23.86 m vs. MCAO: 27.83 ± 13.80 m, *p* = 0.0346), movement duration (Fig. 4c; Non-stroked: 230.55 ± 38.87 s vs. MCAO: 85.87 ± 49.84 s, *p* = 0.0022), and velocity (Fig. 4d; Non-stroked: 0.13 ± 0.04 m/s vs. MCAO: 0.05 ± 0.02 m/s, *p* = 0.0350) compared to the non-stroked group. By 8d post-MCAO, there were no significant differences between groups (*p* > 0.05).

A pig mRS assessment was used to evaluate the degree of disability pre- and post-MCAO with 0 being no residual stroke symptoms and 6 being death (Supplementary Fig. 6). At all timepoints non-stroked animals had an mRS score of 0. At 4 h (h) to 3d post-MCAO, stroked animals had mean mRS scores above 3 (ranging from 2–5) and were significantly impaired relative to non-stroked animals (4 h post-MCAO: 4.86 ± 0.38, *p* < 0.0001; 8 h post-MCAO: 4.57 ± 0.79, *p* < 0.0001; 12 h post-MCAO: 3.71 ± 0.76, *p* = 0.0002; 16 h post-MCAO: 3.71 ± 0.95, *p* = 0.0007; 20 h post-MCAO: 3.57 ± 0.98, *p* = 0.001; 24 h post-MCAO: 3.57 ± 0.79, *p* = 0.0003; 2d post-MCAO: 3.43 ± 0.98, *p* = 0.0013; 3d post-MCAO: 3.29 ± 0.76, *p* = 0.0004). At 4d (2.57 ± 1.13, *p* = 0.0144), 6d (1.71 ± 0.95, *p* = 0.0455) and 8d (1.57 ± 0.79, *p* = 0.0275) post-MCAO, stroked animals still had a significantly greater mRS score than non-stroked controls suggesting persistent deficits. After 8d post-MCAO, scores trended between 0–2 and displayed no significant differences from normal animals (*p* > 0.05) suggesting overall recovery.

### Location of stroke lesion in structures associated with motor coordination had prognostic value when evaluating motor functional outcomes

Development of an accurate prognosis method by identification of acute parameters is vital to assess efficacy of novel treatments in preclinical models. Thus, correlation analyses were performed to determine unique relationships between individual lesioned brain structures at 1d post-MCAO and functional gait outcomes at 2d and 8d post-MCAO. Lesioned structures ranked by PoS (y-axis) and top gait outcomes (x-axis) were evaluated by Pearson correlations for 2d post-MCAO (Fig. 5a) and 8d post-MCAO (Fig. 5b). R values were reported with cell color indicating correlation direction and strength. FDR p-values corrected for multiple comparisons can be found in Supplementary Table 3. In general, structures associated with motor coordination and function had higher R values when using PoS as the quantitative indicator. This suggests that identification of lesion topology, specifically the percentage of structure with an ischemic lesion, can be used as a predictive biomarker for functional outcomes.

Further, commonly used MRI parameters including lesion volume, midline shift, and hemispheric swelling were evaluated for their potential to predict 6 gait functional outcomes (Fig. 5). Pearson correlation analyses were also conducted at 2d and 8d post-MCAO between canonical MRI metrics and functional gait outcomes. For 1d post-MCAO MRI parameters (y-axis) and top gait outcomes (x-axis) were evaluated by Pearson correlations for 2d post-MCAO (Fig. 5c) and 8d post-MCAO (Fig. 5d). R values were reported with cell color indicating correlation direction and strength. FDR p-values corrected for multiple comparisons can be found in Supplementary Table 3. Traditional MRI diagnostic parameters showed moderate prognostic value in this preclinical model. However, the inclusion of lesion topology may further advance the relationship between individual structures and functional outcomes.

## Discussion

MRI assessment of stroke severity and injury characteristics has significant clinical potential to better predict functional outcomes. However, standard clinical practice typically relies on basic injury metrics such as lesion volume and midline shift and does not account for injury location^6,14,40–42^. Since brain functions such as motor and executive control are highly regionalized to specific brain structures, it is logical that consideration of injury location would have a profound effect on clinical prognoses^6^. In this exploratory study, we demonstrated that the canonical MRI metrics (e.g., lesion volume, lesion percent, midline shift, and hemispheric swelling) were predictive of functional deficits. However, we showed that specific lesioned brain structures responsible for motor coordination and function (e.g., putamen, globus pallidus, and primary somatosensory cortex) demonstrated higher R values (R =|0.58| to |0.81|) to motor function deficits than canonical MRI metrics (R =|0.30| to |0.69|). The results from this exploratory study showed that lesion topology analysis may be a stronger predictor of functional outcomes at acute timepoints after ischemic stroke when the structures lesioned involve motor coordination and function. However, additional studies are needed with increased animal numbers to confirm this finding. In recent studies, our team has shown the pig MCAO model displays significant similarities to human stroke patients with respect to functional deficits^29–32,34,37,43^. For the first time in a neural injury porcine model, we have adapted an MRI pig atlas^23^ to account for stroke induced structural changes and demonstrated that the effected MCA territory and brain structures are also comparable to humans, which likely explains the similarities in functional outcomes^44,45^. These results further support the pig as a translational large animal model for the study of ischemic stroke pathophysiology, development of prognostic biomarkers, and therapeutic interventions.

The MCA is one of the largest arteries in the brain with the highest incidence of occlusion causing ischemic stroke in humans^1,44^. Anatomically, the MCA provides vascular support to a large territory including the lateral inferior frontal lobe, superior temporal gyrus, primary motor and somatosensory cortices, basal ganglia structures, internal capsule, and insular cortex, among others^1,2,44^. Thus, the blockage of this artery by an embolus or thrombus leading to an ischemic stroke can be particularly detrimental causing behavioral and gait deficits^18^. Clinically, an MCAO stroke lesion typically spans parts of the frontal, temporal, and parietal lobes, including the insular cortex, as well as deep brain structures such as the amygdala, caudate nucleus, putamen, globus pallidus, and thalamic nuclei^6,14–18,45–47^. In pigs, we found MCAO resulted in the highest percent of lesion in the insular cortex, somatosensory cortices, temporal gyri, and visual cortices. In a thromboembolic MCAO cynomolgus monkey model, infarction was similarly observed in the basal ganglion, internal capsule, temporal cortex, and insular cortex and it was noted that contralateral hemiparesis in their model was likely caused by ischemia in the temporal cortex and internal capsule^48^. Although the MCA stroke territory depends on the individual differences of MCA branching, our model showed a high level of reproducibility with respect to infarct location. 100% of the pigs showed lesioning in the claustrum, inferior temporal gyrus, insular cortex, parahippocampal cortex, putamen, prepiriform area, secondary visual cortex, and superior temporal gyrus. Thus, we have identified consistent and reproducible lesion locations that can aid in the understanding of the pathophysiology of stroke progression and provide a framework for assessing the importance of lesion location on functional outcomes.

Gait and behavior analyses have been used as clinical biomarkers to quantify stroke severity, functional deficits, and subsequent recovery potential. Consistent with human manifestations where patients have decreased cadence and walk six times slower than aged matched pairs^35^, the porcine MCAO group showed similar deficits in cadence and velocity^35,36^. Humans display other spatiotemporal abnormalities including a decreased stride length, increased cycle time, and increased stance time^49^, which have consistently manifested in the porcine model^30,32,37^. Asymmetrical hemiplegic gait is commonly induced by stroke and results in a shifting of weight between limbs and subsequent decreased propulsion, where the contralateral limb assumes significantly less weight and decreased pressure^36^. Here, stroked pigs showed asymmetrical hemiplegic gait with a decreased total pressure index in the contralateral limb. As seen in the open field test and mRS scoring, MCAO pigs displayed deficits similar to those of stroke patients, including decreased voluntary movement and velocity, with overall behavioral deficits^50,51^. These impairments are typically a result of infarction to the motor and somatosensory cortices, and subcortical structures^50^, similar to the regions injured in our pig model. Manifestation of clinically relevant gait deficits in the porcine model suggests that this model may be useful in understanding post-stroke functional deficits and recovery patterns.

Recent clinical retrospective analyses assessing the prognostic capacity of early MRI stroke injury parameters indicate lesion location can be highly predictive of functional outcomes, whereas lesion volume alone is only moderately predictive^4,12–14,17,52^. In fact, a voxel-based lesion mapping approach showed location, independent of lesion volume, is predictive of higher NIHSS scores at admission and discharge^18^. Here, regional lesion location showed higher R values to acute functional outcomes in brain regions responsible for motor function, including the putamen, globus pallidus, and primary somatosensory cortex, at 2d post-MCAO compared to brain regions that are not responsible for motor coordination but still highly effected by the stroke lesion, including the claustrum, superior and middle temporal gyri, and secondary visual cortex. The structures impacted that presented higher correlations to gait outcomes are directly involved with motor and executive control and therefore it is expected that they would influence gait deficits 2d post-MCAO^53–55^. For example, effected structures, such as the globus pallidus (a critical regulatory structure of voluntary movement^56^), were predictive of early functional outcomes such as velocity with an R value of  − 0.76 at 2d post-MCAO, whereas lesion volume only had an R value of  − 0.37. In addition, the insular cortex, which is sensitive to hypoperfusion^47^, has a high likelihood of infarct growth^10^, and is associated with functional deficits^46^, was affected in 100% of pigs at 1d post-MCAO and was the largest PoL structure effected. The insular cortex also displayed moderate correlation relationships between PoS and gait impairments (R =|0.37| to |0.56|). In a large clinical study, MCA strokes resulted in higher disability with the lowest functional independence measure (FIM), a functional assessment that consists of 13 motor and 5 cognitive metrics, compared to other territory strokes^57^. These findings support strong correlations between MCA territory lesioning of structures involved in motor coordination and motor deficits. Many clinical studies often examine the correlation between MRI based structural changes and one or two functional scales, such as mRS or NIHSS scores^7,18,57–59^, while our study evaluated the relationship between multiple major gait parameters acquired on the sensitive GAITFour mat and the highly lesioned structures in our porcine model. Here, we found that ischemic infarcts in the putamen, globus pallidus, and primary somatosensory cortex were associated with higher R values to motor functional deficits (R >|0.5|). A voxel-based lesion symptom mapping approach in humans determined infarcts within the area leading from the corticospinal tract to cortical motor areas, including fibers from the primary somatosensory cortex and other secondary motor regions, were critical for maintaining proper hand motor performance after a stroke^60^. We were encouraged to discover similar relationships between major structures responsible for motor function and functional gait outcomes in our pig model.

However, a number of limitations were associated with this exploratory study including the lack of significance in our correlation analyses, although it is likely a function of small sample size (Supplementary Table 3). Additionally, correlations between MRI and motor function at 8d post-MCAO were reduced relative to 2d post-MCAO. This may be a result of spontaneous recovery observed in functional outcomes 8d post-MCAO, even though significant differences still remained between stroked and non-stroked animals. A general shortcoming of the topological analysis approach is each structure should be considered individually based on its function and associated outcomes. This adds an increased level of complexity with the evaluator needing to be well versed in the function of each structure and making it less practical as a clinical prognostic tool. A benefit of the traditional MRI analysis approaches (e.g., lesion volume, midline shift, and swelling) is the ease of clinical use with no neuroanatomical key of functional information. Furthermore, in both animal stroke models and clinical patients, MRI image registration to neuroanatomical atlases are often imperfect due to hemispheric swelling and atrophy. In our pig model, registration of larger structures demonstrated an acceptable level of atlas registration fidelity, while there was decreased accuracy in the registration of smaller structures such as the contralateral ventricle and small white matter structures. These potential inconsistencies represent an unmet need in the preclinical and clinical settings to improve atlas registration techniques accounting for swelling, midline shifts, and other commonly observed stroke pathologies. The rodent community has made strides to account for registration errors including the development of an enhanced mathematical framework to correct for edema in evaluating lesion size leading to improved image analysis^61^. Future studies utilizing the pig stroke model should build upon these advances to improve registration. Despite these limitations, the topological approach utilized in this study has helped identify lesioned structures involved in motor function related to stroke outcomes and supports the clinical use of this methodology. Further in-depth studies with larger cohorts are warranted to generate more conclusive preclinical data.

In this exploratory study utilizing the Yucatan pig MCAO model, we demonstrated that evaluating lesioning in specific brain structures demonstrated moderate-to-strong relationships with motor function impairments as compared to lesion volume and other commonly assessed clinical MRI stroke metrics at acute timepoints. The lesioned MCAO territory in the pig model was anatomically similar to human ischemic stroke patients and resulted in comparable functional outcomes, supporting the pig as a robust ischemic stroke model. It is important to note that other key elements such as functional and structural connectivity may also be critical components in predicting functional outcomes and should be further explored in future studies^62–64^. Nevertheless, using stroke location to improve outcome prediction is rapidly evolving in clinical practice. Currently, the use of stroke location to predict functional outcomes is under assessed in animal models that tend to report traditional metrics such as lesion volumes and midline shifts. Increased frequency of the identification of stroke lesion topology correlated to functional outcomes in preclinical animal models may lead to greater clinical translatability. Improving the accuracy and precision of clinical prognoses is critically important in evidence-based clinical decision-making after stroke to improve acute patient care, develop long-term treatment plans (e.g., rehabilitation programs), and determine required living assistance needs. The findings of this study support the premise of including a structural injury component in acute MRI assessment to improve clinical prognostication.

## Materials and methods

### Study design

All work involving the use of animals in this study were performed in accordance with the National Institutes of Health (NIH) Guidelines for the Care and Use of Laboratory Animals and was reviewed and approved by the University of Georgia (UGA) Institutional Animal Care and Use Committee (IACUC; Protocol Number A2018 01-029-Y1-A5). Inclusion criteria established a priori indicated that all castrated/ovariectomized, healthy animals with no lameness would be included. Researchers would exclude an animal if any signs of illness or lameness presented. All animals were included in the study and the experiments have been reported following/in compliance with the Animal Research: Reporting in Vivo Experiments (ARRIVE) guidelines. Biomedical Yucatan miniature pigs were acquired from Exemplar Genetics (Sioux Center, IA) and individually housed. Sexually mature castrated male and ovariectomized (OVX) female Yucatan miniature pigs between 68–98 kg and between 1–2 years of age were randomly assigned to either stroke (MCAO, n = 7) or non-stroked normal control (non-stroked, n = 5) groups. Sex hormones (e.g., testosterone and estrogen) have a confounding effect as they are neuroprotective^65–67^. This challenge is compounded in female animals where phases of the reproductive cycle increase and decrease estrogen levels, thus leading to constantly changing neuroprotective levels in animals. Additionally, strokes typically occur in older women, post-menopause^68^. For uniformity, males were castrated, and females were OVX. All functional analysis was performed blinded with animal identifiers removed. The sample size for this study was determined by a power calculation based on our previous porcine MCAO studies^29,32^.

### Middle cerebral artery occlusion

One day prior to surgery, pigs were administered antibiotics (Ceftiofur crystalline free acid; 5 mg/kg intramuscular (IM); Zoetis). A right sided permanent MCAO was performed on all stroke animals as described in Platt, et al.^69^. Pre-induction analgesia and sedation was achieved using xylazine (4 mg/kg IM; VetOne ), midazolam (0.3 mg/kg IM; Heritage), methadone (0.2 mg/kg IM; Henry Schein Animal Health). Anesthesia was induced with propofol (to effect, intravenous (IV); Zoetis) and prophylactic lidocaine (1.0 mL 2% lidocaine; VetOne) was applied to the laryngeal folds to facilitate intubation. Anesthesia was maintained with isoflurane (1.0–2.0%; Abbott Laboratories) in oxygen.

Briefly, the middle cerebral artery (MCA) was accessed by performing a frontotemporal craniectomy with orbital rim ostectomy, zygomatic arch resection, and temporal fascia and muscle incision. The MCA was permanently occluded utilizing bipolar electrocautery forceps at the location distal to the origin of the Circle of Willis, resulting in ischemic infarction. The exposed brain was covered with a sterile oxidized cellulose hemostatic agent (VetSpon). Following occlusion, the temporalis muscle and epidermis were routinely re-apposed. After surgery, anesthesia was discontinued, pigs were returned to their pens and underwent standard monitoring and pain management. To treat and manage post-operative inflammation and pain, MCAO pigs received banamine (2.2 mg/kg IM or IV; Merck) during MCAO surgery and every 12 h for 24 h and every 24 h for 3 days following surgery and methadone (0.2 mg/kg IM or IV) every 6 h for 24 h following surgery. Non-stroked normal animals did not undergo surgery.

### Magnetic resonance imaging data acquisition

MRI was acquired on a GE Signa HDx 3.0 T scanner using an 8-channel torso coil at 1d and 28d post-MCAO. Under general anesthesia, MRI of the brain was performed with the animal positioned in supine recumbency. The multiplanar MRI protocol included five sequences: 1) 3D Fast SPoiled GRadient echo (FSPGR) T1-Weighted (T1W), 2) Fast Spin Echo (FSE) T2W, 3) T2-Weighted Fluid Attenuated Inversion Recovery (T2FLAIR), 4) Spin Echo (SE) Diffusion Weighted Imaging (DWI), and 5) SE DTI.

Ischemic stroke was confirmed 1d post-MCAO by comparing the hyperintense infarction in T2FLAIR and DWI to the corresponding hypointense region in apparent diffusion coefficient (ADC) maps, indicating cytotoxic edema (data not shown). Axial 3D FSPGR sequences were acquired with the following parameters: inversion time (TI) = 450 ms, flip angle = 20°, slice thickness = 1.0 mm, field of view (FOV) = 19.8 cm^3^, and matrix size (frequency x phase) = 198 × 198. Axial and coronal FSE T2W images were acquired with the following parameters: repetition time (TR) = 6260 ms, echo time (TE) = 124 ms, slice thickness = 3.0 mm, FOV = 18–20 cm^2^, and matrix size (frequency x phase) = 384 × 288. Axial T2FLAIR images were acquired with the following parameters: TR = 9070 ms, TE = 120 ms, TI = 2587 ms, slice thickness = 3.0 mm, FOV = 20 mm^2^, and matrix size (frequency x phase) = 320 × 224. Axial DWI was acquired with the following parameters: b = 0 and 1000, TR = 6000 ms, number of excitation (NEX) = 4, slice thickness = 3.0 mm, FOV = 26.6 cm^2^, matrix size (frequency x phase) = 256 × 256 with 3 diffusion directions. In order to obtain information on white matter integrity, DTI was acquired in the axial plane with the following parameters: TR = 10,000 ms, isotropic voxel = 2.0 mm × 2.0 mm × 2.0 mm, with 30 diffusion encoding directions; fractional anisotropy (FA) maps were generated from DTI images as described below. During DTI acquisition, an error occurred when acquiring the DTI sequence of one MCAO animal, therefore, this animal was excluded from FA analysis.

### Magnetic resonance imaging data preprocessing

DICOM images of the T1W and T2W volumetric series were converted into NIfTI (Neuroimaging Informatics Technology Initiative) format using the “dcm2niix” tool^70^. Brain masks were obtained by separating brain tissue from the skull and other surrounding tissues using the FSL brain extraction tool (BET) and then manually refining slice-by-slice the generated FSL-BET masks in 3D Slicer version 4 (slicer.org^71^). Lesion masks were manually drawn in 3D Slicer using the axial T2W images to identify the hyperintense region associated with the lesion after applying an inversion filter to remove bias in lesion identification (Supplementary Fig. 1).

The porcine brain atlas^23^ was then spatially normalized to each stroked pig’s masked T1W image (note that each stroked pig’s T2W image is in the same space as its T1W image). First, a spatial transformation was calculated between the pig brain atlas’ associated T1W anatomical image, which is in the same space as the atlas, and each pig’s masked T1W image using the Old Normalize Statistical Parametric Mapping algorithm (SPM12, Institute of Neurology, University College London)^72^. Then the calculated spatial transformation was applied to the atlas. Spatial transformations consisted of a 12-parameter affine transformation, followed by a nonlinear deformation transformation in stereotaxic coordinates^73,74^.

### Magnetic resonance imaging data analysis

Lesion and hemispheric volumes were analyzed using OsiriX software (Version 10.0.5, Pixmeo SARL, Bernex, Switzerland) at default thresholds from T2W sequences. Hemisphere and lateral ventricle volumes were calculated for each slice using manual segmentation of the ipsilateral and contralateral hemispheres to find the area and multiplying by the slice thickness (3.0 mm). The lateral ventricle volumes were subtracted from each hemisphere to eliminate non-brain tissue. The cerebellum was not included in hemispheric volume analysis.

The percentage of the ipsilateral hemisphere that the lesion occupied was calculated as a sum of the lesion volume per slice (multiplied by slice thickness, 3.0 mm) divided by the total ipsilateral hemispheric volume.

Hemispheric swelling and atrophy were calculated using the total ipsilateral hemisphere volume divided by the contralateral hemisphere volume, which is reported as a percentage change.

Midline shift analysis was also analyzed using OsiriX according to a previous publication^34^. Briefly, three linear measurements were utilized to determine the midline after stroke at 1d and 28d post-stroke using the axial T2W images for each pig. The first line was drawn at the septum pellucidum in between the lateral ventricles. The next line drawn was the ideal midline using boney structures and non-brain anatomy to determine the natural midline (red lines in Fig. 1). The midline shift was determined by drawing and measuring a third perpendicular line between the ideal midline and septum pellucidum.

DTI was used to evaluate white matter integrity in the corpus callosum (CC) through FA analysis. FA maps were generated using the FMRIB’s Diffusion Toolbox (FDT) (FSL, University of Oxford, UK)^75–77^, and FA values of the CC were measured using ImageJ, FIJI^78,79^ to manually segment each major white matter tract in the ipsilateral and contralateral hemispheres. To account for hemispheric swelling and atrophy, the ipsilateral ROI was manipulated to encompass as much of the remaining visible structure possible without changing the area of the ROI.

Using the co-registered lesion masks and porcine brain atlas^23^, percentage of structure (PoS) and percentage of lesion (PoL) were calculated using MatLab (R2018b, The Mathworks, Inc.) to determine the percentage of each individual structure affected by the lesion and the percentage of the lesion that overlaps with individual brain atlas structures, respectively.

$$ {\text{PoS}} = \frac{Number\, of\, pixels\, in\, the\, identified\, lesion\, that\, overlap\, with\, structure\, X}{{Total\, number\, of\, pixels\, within\, structure\, X}} $$

$$ {\text{PoL}} = \frac{Number\, of\, pixels\, in\, the\, identified\, lesion\, that\, overlap\, with\, structure\, X}{{Total\, number\, of\, pixels\, within\, the\, lesion}} $$

T1W images were used to generate figures. The pig brain atlas contained 178 individual cerebral structures, including 42 paired and 9 single deep brain structures, 5 ventricular system areas, 6 paired deep cerebellar nuclei, 12 cerebellar lobules, and 28 cortical areas per hemisphere^23^.

### Gait data collection

Gait analysis was performed utilizing the GAITFour walkway system as previously described^32^ and assessed using GAITFour software (Version 4.9 × 5, CIR Systems, Franklin, NJ). Pre-MCAO gait data was collected on 3 separate days (d) for each pig. After stroke surgery, gait analysis was performed on 2d, 8d, 15d, and 27d post-MCAO. Gait collection occurred for a maximum of 15 min at each collection time point for each pig. Five recordings of the pig traversing the track at a consistent trotting gait was used for analysis.

### Behavior data collection

Open field testing was performed for all pigs pre-MCAO and on 2d, 8d, 15d, and 27d post-MCAO. Behavior tests were recorded using EthoVision XT software (Version 11.5, Noldus Systems, Wageningen, Netherlands) as described previously^32^. Briefly, individual pigs were placed in a 2.7 × 2.7 m open field arena for 10 min and allowed to voluntarily explore. EthoVision was used to track movement duration, velocity, and distance traveled and also to preform data analysis.

### Modified rankin scale scoring

Neurological disability was assessed post-MCAO using a pig-adapted Modified Rankin Scale (mRS) as previous published^34^. All pigs were assessed 1 day prior to MCAO surgery and 4 h (h), 8 h, 12 h, 16 h, 20 h, 24 h, 2d, 3d, 4d, 5d, 6d, 8d, 15d, and 27d post-MCAO surgery. Possible scores ranged from 0 (no residual stroke symptoms) to 6 (death due to stroke).

### Statistical analysis

GraphPad Prism 8 (Version 8.4.0; San Diego, CA) was utilized to calculate the statistics. Statistical differences in MRI parameters were evaluated using paired t tests between 1 and 28d post-MCAO. White matter integrity was evaluated between the left and right CC with paired t tests at pre-MCAO, 1d and 28d post-MCAO. To determine if MLS and hemispheric swelling/atrophy were statistically different from normal at each timepoint, one-sample t tests were performed with a hypothetical value of 0 for MLS and 100 for hemispheric changes, statistical significance was indicated by $. For longitudinal analyses of gait and behavior, 2-way repeated measures ANOVA were conducted with Sidak’s multiple comparisons test when appropriate. All error bars represented SD. Pearson correlations were evaluated at 2d and 8d post-MCAO for the 6 gait parameters reported in the manuscript to determine the association between percent of lesioned structure (top reported PoS) and canonical MRI metrics (lesion volume, lesion percent, midline shift, and hemispheric swelling) to motor impairments. A minimum of three pigs had to have a lesion within the individual structure to conduct each correlation analysis. From here, R values were reported in a heat map and the False Discovery Rate (FDR) corrected p-values were reported in Supplementary Table 3. QQ plots of the residuals were generated to assess normality. Data are shown as mean ± SD. Statistical significance was indicated where * (or # or $) signified *p* < 0.05; ** (or ## or $$) signified *p* < 0.01; *** (### or $$$) signified *p* < 0.001; and **** (or #### or $$$$) signified *p* < 0.0001.

## Supplementary information

Supplementary information.

## Data Availability

Data and MatLab code is available at https://zenodo.org/.
