# Supplementary material for: Exploring the predictive value of lesion topology on motor function outcomes in a porcine ischemic stroke model
Source: Sci Rep. 2021 Feb 15;11:3814. doi: 10.1038/s41598-021-83432-5 (PMC7884696; doi:10.1038/s41598-021-83432-5)
Supplement: Supplementary file 1 — Supplementary information. [file 41598_2021_83432_MOESM1_ESM.pdf]

Exploring the predictive value of lesion topology on motor function outcomes in a porcine ischemic stroke model

Kelly M. Scheulin[1-3]#, Brian J. Jurgielewicz[1-3]#, Samantha E. Spellicy[1-3], Elizabeth S. Waters[1-3], Emily W. Baker[5], Holly A. Kinder[1,2], Gregory A. Simchick[1,4], Sydney E. Sneed[1,2], Janet A. Grimes[6], Qun Zhao[1,4], Steven L. Stice[1,2,3,5], Franklin D. West[1,2,3]*.

# These authors contributed equally to this work

Author Affiliations:

[1]University of Georgia, Regenerative Bioscience Center, Athens, GA, USA

[2]University of Georgia, Department of Animal and Dairy Sciences, Athens, GA, USA

[3]University of Georgia, Biomedical and Health Sciences Institute, Neuroscience Program, Athens, GA, USA

[4]University of Georgia, Department of Physics, Athens, GA, USA

[5]Aruna Bio Inc, Athens, GA, USA

[6]University of Georgia, College of Veterinary Medicine, Department of Small Animal Medicine and Surgery, Athens, GA, USA

* Corresponding Author: 425 River Road RM 316, Athens, GA 30602; Email: WestF@uga.edu; Phone: 706-542-0988

Supplementary Information

Exploring the predictive value of lesion topology on motor function outcomes in a porcine ischemic stroke model

Supplementary Tables and Figures

Supplementary Table 1: Percent of Structure and Percent of Lesion 1d and 28d post-MCAO

	**Percent of Structure 1d post-MCAO**		**Percent of Structure 28d post-MCAO**		**Percent of Lesion 1d post-MCAO**		**Percent of Lesion 28d post-MCAO**	
**Structure**	**Mean**	**SD**	**Mean**	**SD**	**Mean**	**SD**	**Mean**	**SD**
Amygdala	24.13	27.18	4.31	8.25	1.23	1.95	0.60	1.03
Anterior Commissure	0.97	2.55	0	0	0.004	0.01	0	0
Anterior Entorhinal Cortex	9.95	9.29	0.16	0.43	0.93	1.66	0.06	0.15
Anterior Prefrontal Cortex	2.43	4.40	0	0	0.19	0.36	0	0
Associative Visual Cortex	15.49	13.95	10.02	14.48	0.54	0.51	0.85	1.03
Caudate Nucleus	7.20	8.73	0	0	0.45	0.62	0	0
Claustrum	65.91	29.68	0.96	2.45	1.94	0.71	0.07	0.17
Corpus Callosum	0.22	0.56	0	0	0.03	0.07	0	0
Dorsal Anterior Cingulate Cortex	0.19	0.51	0	0	0.008	0.02	0	0
Dorsolateral Prefrontal Cortex	1.23	2.05	0.01	0.03	0.07	0.12	0.002	0.006
Fornix	2.93	2.82	0.25	0.53	0.18	0.18	0.08	0.18
Fusiform Gyrus	10.43	12.06	6.82	10.44	0.72	1.31	0.59	0.77
Geniculate Nucleus	0.04	0.12	0	0	0.0004	0.001	0	0
Globus Pallidus	34.52	28.61	0	0	0.59	0.69	0	0
Hippocampus	4.46	3.66	0.46	1.23	0.48	0.68	0.21	0.54
Inferior Temporal Gyrus	62.15	27.67	18.19	13.55	5.16	4.65	3.27	2.72
Insular Cortex	54.45	31.35	15.95	9.28	8.45	5.09	7.95	6.02
Middle Temporal Gyrus	44.43	24.49	39.84	31.80	2.59	2.97	4.23	3.01

Parahippocampal Cortex	12.98	10.99	3.11	3.51	2.06	3.00	1.42	2.34
Piriform Cortex	0	0	0.21	0.54	0	0	0	0
Prepiriform Area	23.76	15.86	7.84	5.85	1.44	1.31	1.30	1.06
Primary Motor Cortex	0.18	0.32	0	0	0.007	0.01	0	0
Primary Somatosensory Cortex	33.48	23.73	8.44	8.92	6.33	4.82	5.65	6.36
Primary Visual Cortex	4.90	6.86	0.02	0.03	1.08	1.36	0.009	0.02
Pulvinar Nuclei	0.53	0.98	0	0	0.008	0.02	0	0
Putamen	44.00	22.23	0.27	0.72	3.22	1.58	0.04	0.11
Reticular Thalamic Nucleus	7.61	9.74	0	0	0.09	0.13	0	0
Orbitofrontal Cortex	0.41	1.08	0	0	0.002	0.004	0	0
Secondary Visual Cortex	40.57	28.62	5.83	6.30	6.02	4.34	2.07	1.93
Somatosensory Association Cortex	43.05	27.06	16.78	11.46	4.98	2.87	5.41	3.59
Subiculum	2.23	3.56	0	0	0.02	0.03	0	0
Superior Temporal Gyrus	58.91	36.32	31.14	17.55	2.36	1.68	3.15	1.87
Ventral Anterior Thalamic Nucleus	0.02	0.06	0	0	0.001	0.002	0	0
Ventral Posterior Thalamic Nucleus	0.29	0.40	0	0	0.004	0.006	0	0

Supplementary Table 1: Percent of structure signifies the percent of individual anatomical structure with a lesion. Percent of structure (PoS) was calculated by the total number of activated pixels (lesion) in structure divided by the total number of pixels in structure. Percent of lesion (PoL) signifies the percent of each structure in the identified lesion. PoL was calculated by dividing number of activated pixels per structure by the total number of pixels of the lesion.

Supplementary Table 2: Table of Functional Parameters Descriptions

	**Gait or Behavior Parameter Measured**
Spatial Gait Parameters	▪ Step Length (cm) LF, RF, LH, RH (*Measured between the heel center of the current hoof print to the heel center of the previous hoof print on the opposite foot*)<br>▪ Stride Length (cm) LF, RF, LH, RH (*Distance between consecutive hoof prints of the same hoof)*
Temporal Gait Parameters	▪ Velocity (*Distance traveled (cm)/sec*)<br>▪ Cadence (S*trides/min*)<br>▪ Swing Percent (%) LF, RF, LH, RH *(The percent of 1 full gait cycle in which the limb was in the non-contact phase*)<br>▪ Stance Percent (%) LF, RF, LH, RH *(The percent of 1 full gait cycle in which the limb was in the contact phase*)
Pressure Gait Parameters	▪ Total Pressure Index LF, RF, LH, RH (*TSP expressed as a percentage of all four limbs. This shows % of weight distribution across all four hoofs*)
Open Field Mobility Behavior Parameters	▪ Distance (m) (*Distance traveled by the pig within the open field arena)*<br>▪ Velocity (*Distance traveled (cm)/sec*)<br>▪ Movement Moving Duration (*Elapsed time the running average velocity exceeds the start velocity*)
Modified Rankin Scale Score	Observational behavior assessment modified for a pig. 0 (no residual stroke symptoms) to 6 (death due to stroke). *See Spellicy et al.*[1] *for further information on the scale.*

Supplementary Table 2: Functional outcomes evaluated. Compiled list of functional outputs with corresponding definitions.

Supplementary Table 3: FDR Corrected P-Values

	2d post-MCAO					
	Velocity	Cadence	Step Length LF	Stride Length LF	Swing % LF	Stance % LF
Claustrum	0.8134	0.8134	0.8134	0.8134	0.8134	0.8134
Inferior Temporal Gyrus	0.5704	0.5704	0.5704	0.5704	0.5704	0.5704
Superior Temporal Gyrus	0.6893	0.6893	0.6893	0.6893	0.9716	0.9716
Insular Cortex	0.4716	0.4716	0.4716	0.4716	0.4716	0.4716
Middle Temporal Gyrus	1	1	1	1	1	1
Putamen	0.2336	0.2336	0.2336	0.2336	0.2336	0.2336
Somatosensory Association Cortex	0.5156	0.5156	0.5156	0.5156	0.5156	0.5156
Secondary Visual Cortex	0.775	0.775	0.775	0.775	0.775	0.775
Globus Pallidus	0.1592	0.1592	0.1592	0.1933	0.1978	0.1978
Primary Somatosensory Cortex	0.2211	0.2211	0.2211	0.2211	0.2211	0.2211

	8d post-MCAO					
	Velocity	Cadence	Step Length LF	Stride Length LF	Swing % LF	Stance % LF
Claustrum	0.6721	0.6721	0.6721	0.6721	0.6721	0.6721
Inferior Temporal Gyrus	0.9846	0.9846	0.9846	0.9846	0.9846	0.9846
Superior Temporal Gyrus	0.9712	0.9712	0.9712	0.9712	0.9712	0.9712
Insular Cortex	0.9026	0.9026	0.9026	0.9026	0.9026	0.9026
Middle Temporal Gyrus	0.9592	0.9592	0.9592	0.9592	0.9592	0.9592
Putamen	0.7074	0.7074	0.7074	0.7074	0.7074	0.7074

Somatosensory Association Cortex	0.7547	0.7547	0.7547	0.7547	0.7547	0.7547
Secondary Visual Cortex	0.6198	0.6198	0.6198	0.6198	0.6198	0.6198
Globus Pallidus	0.9173	0.9173	0.9173	0.9173	0.9173	0.9173
Primary Somatosensory Cortex	0.9879	0.9879	0.9879	0.9879	0.9879	0.9879

	2d post-MCAO					
	Velocity	Cadence	Step Length LF	Stride Length LF	Swing % LF	Stance % LF
Lesion Volume	0.5356	0.5356	0.5356	0.5356	0.5356	0.5356
Lesion Percent	0.4492	0.4492	0.4492	0.4492	0.4492	0.4492
Midline Shift	0.3648	0.3648	0.3648	0.3648	0.3648	0.3648
Swelling	0.5673	0.5673	0.5673	0.5673	0.5673	0.5673

	8d post-MCAO					
	Velocity	Cadence	Step Length LF	Stride Length LF	Swing % LF	Stance % LF
Lesion Volume	1	1	1	1	1	1
Lesion Percent	0.9574	0.9574	0.9574	0.9574	0.9574	0.9574
Midline Shift	0.6735	0.6735	0.6735	0.6735	0.6735	0.6735
Swelling	0.7248	0.7248	0.7248	0.7248	0.7248	0.7248

Supplementary Table 3: FDR Corrected P-Values. False discovery rate analysis was completed on original p-values to correct for multiple comparisons.

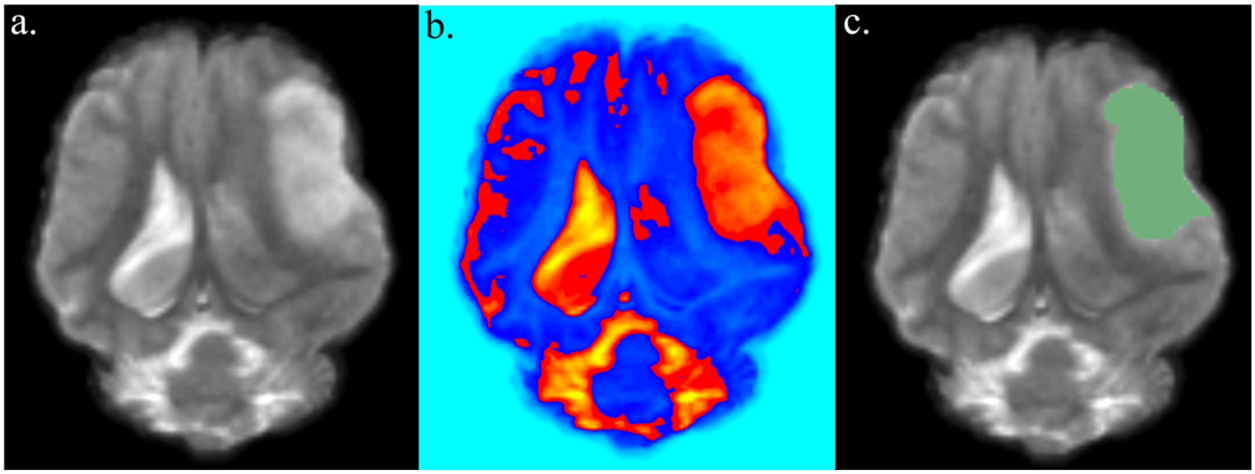

Supplementary Figure 1: Method of identifying lesion volume with inversion filter. T2W image (**a**) with a territorial hyperintense lesion in the ipsilateral hemisphere. Inversion filter applied (**b**) and lesion manually outlined in each slice (**c**).

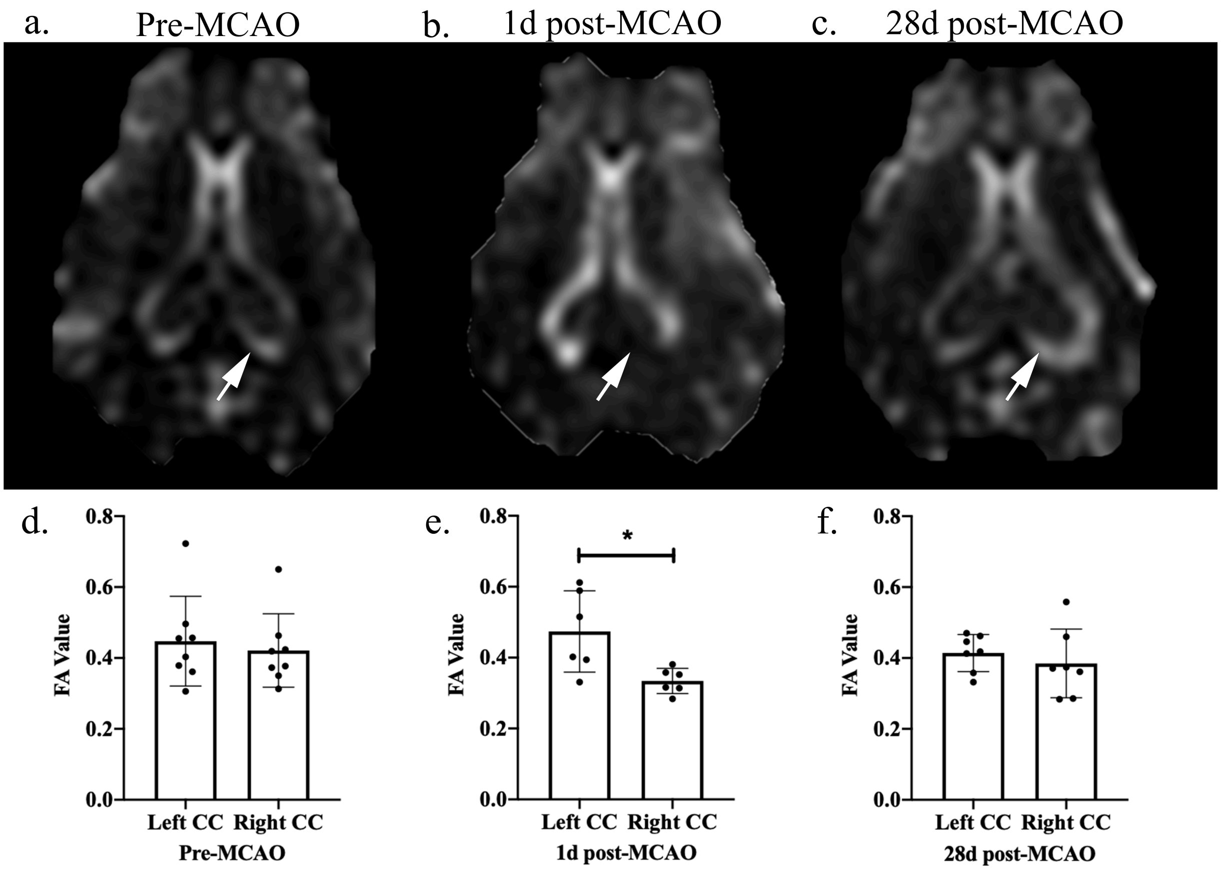

Supplementary Figure 2: MCAO results in acute white matter integrity decreases in the corpus callosum. Axial DTI images show an intact corpus callosum (CC) at pre-MCAO (**a**), reduced ipsilateral (right CC) integrity at 1d post-MCAO (**b**), and recovered ipsilateral integrity at 28d post-MCAO (**c**). Pre-MCAO fractional anisotropy (FA) values did not reveal a significant difference between hemispheres (**d**). At 1d post-MCAO, ipsilateral (right) CC FA value was reduced compared to the contralateral (left) CC (**e**). By 28d post-MCAO, FA values had recovered (**f**). * indicated statistical difference between hemispheres.

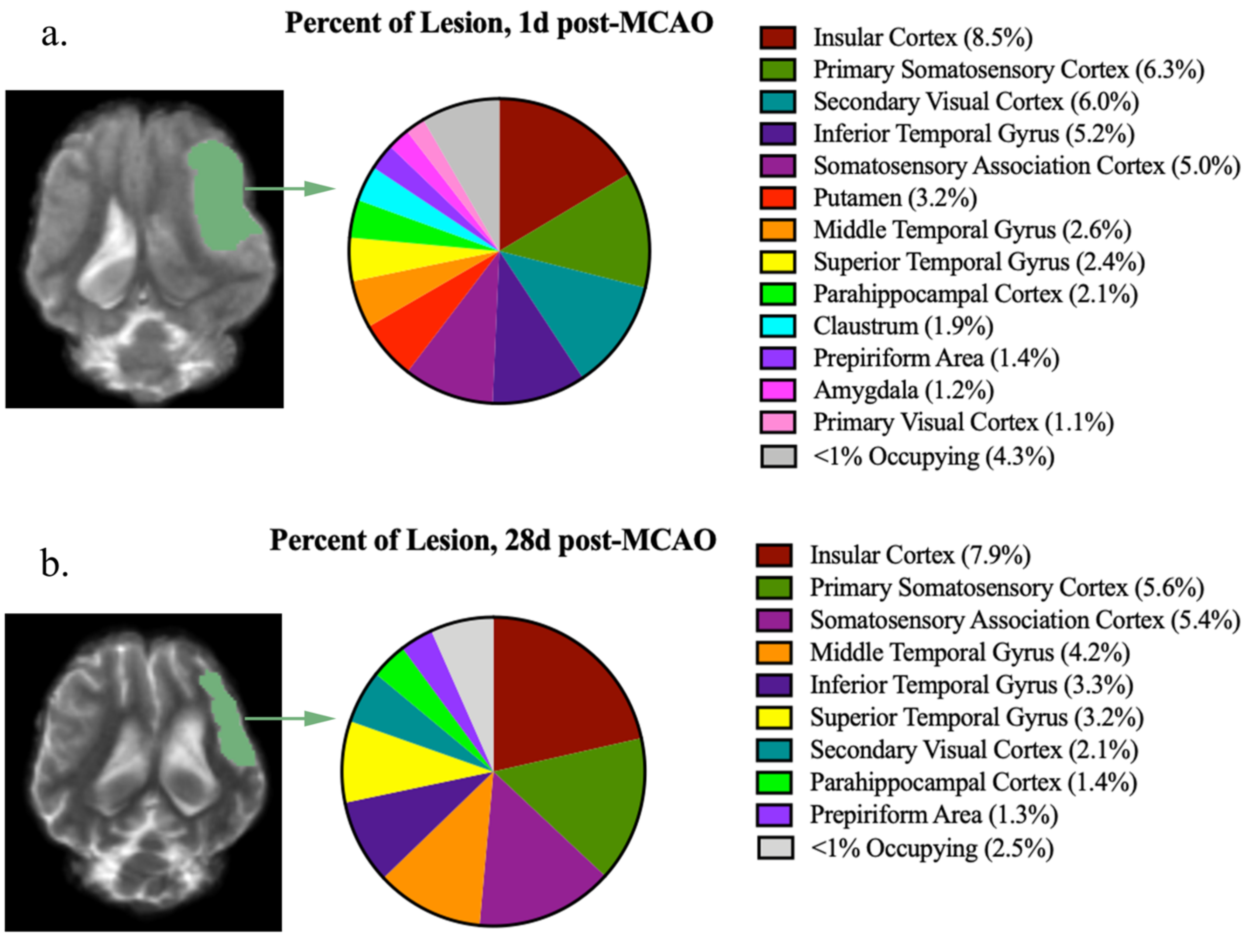

Supplementary Figure 3: Percent of identified lesion in effected brain structures. The percent of the identified lesion (PoL) in each structure was quantified to determine the percent of the identified lesion that affected specific brain structures. Summary of the structures occupying the highest percent of lesion measured at 1d post-MCAO (**a**) and at 28d post-MCAO (**b**).

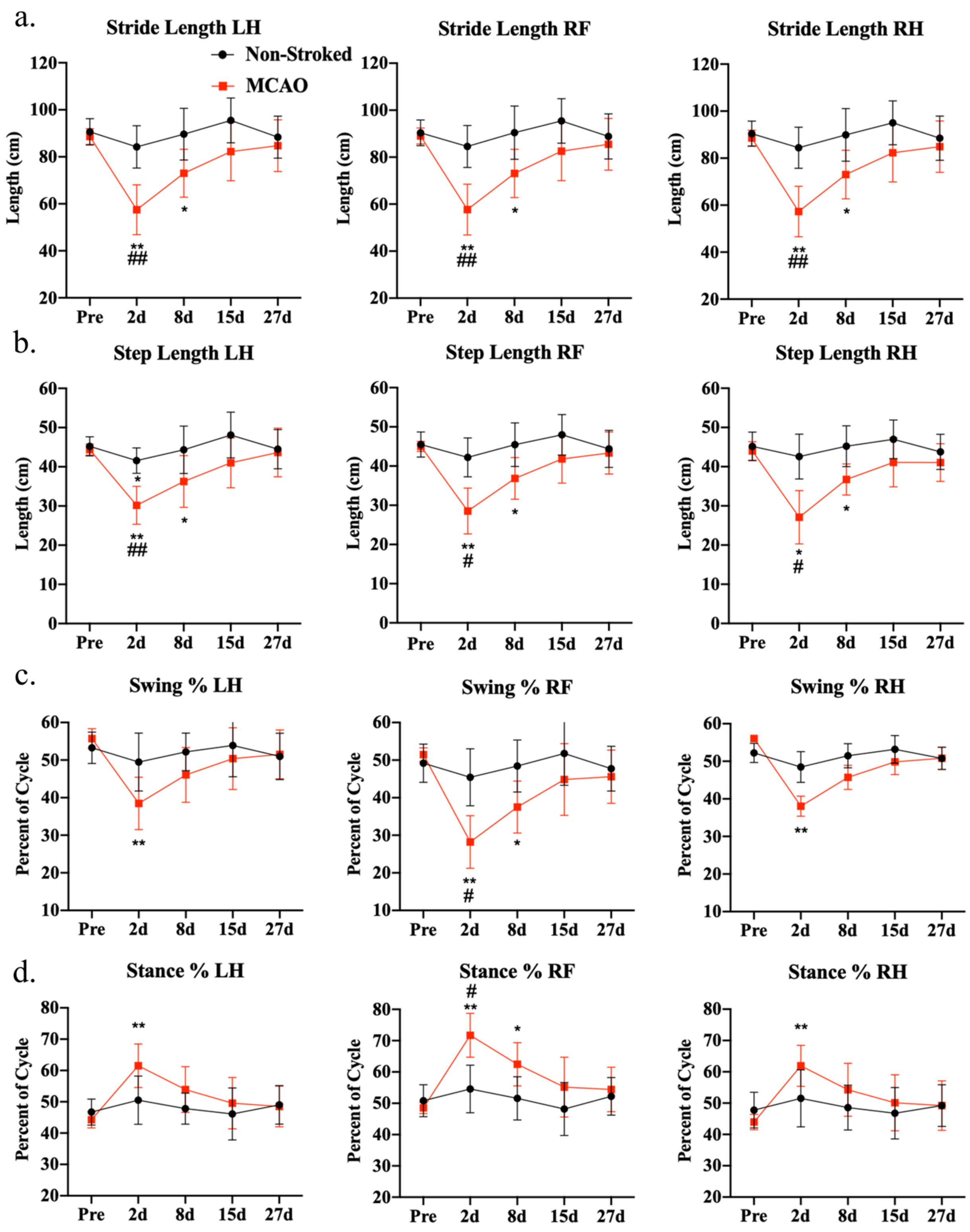

Supplementary Figure 4: MCAO resulted in acute deficits in right front, right hind, and left hind spatiotemporal gait parameters. Stride length of the left hind (LH), right front (RF),

and right hind (RH), decreased at 2d (p=0.0038, p=0.0037, and p=0.0040, respectively) and 8d (p=0.0290, p=0.0277, and p=0.0325, respectively) post-MCAO compared to pre-MCAO values (**a**). Stroked animals exhibited a decrease in stride length at 2d post-MCAO compared to non-stroked controls in LH (p=0.0075), RF (p=0.0073), and RH (p=0.0063). Step length of the LH, RF, and RH decreased at 2d (p=0.0017, p=0.0050, and p=0.0104, respectively) and 8d (p=0.0437, p=0.0332, and p=0.0238, respectively) post-MCAO compared to pre-MCAO values (**b**). Stroked animals exhibited a decrease in step length at 2d post-MCAO compared to non-stroked controls in LH (p=0.0067), RF (p=0.0117), and RH (p=0.0135). Swing percent decreased in the LH (p=0.0093), RF (p=0.0033), and RH (p=0.0091) at 2d post-MCAO and in the RF (p=0.0110) at 8d post-MCAO compared to pre-MCAO values (**c**). Stroked animals exhibited a decrease in swing percent at 2d post-MCAO compared to non-stroked controls in RF (p=0.0219). Stance percent increased in the LH (p=0.0093), RF (p=0.0033), and RH (p=0.0091) at 2d post-MCAO and in the RF (p=0.0110) at 8d post-MCAO compared to pre-MCAO values (**d**). Stroked animals exhibited an increase in stance percent at 2d post-MCAO compared to non-stroked controls in RF (p=0.0222). Non-stroked controls had no changes in the measured parameters for the duration of the study (p>0.05), except for step length LH compared to pre-MCAO values at 2d post-MCAO (p=0.0497). * and ** indicated statistical difference from pre-MCAO values. # and ## indicated statistically different between groups.

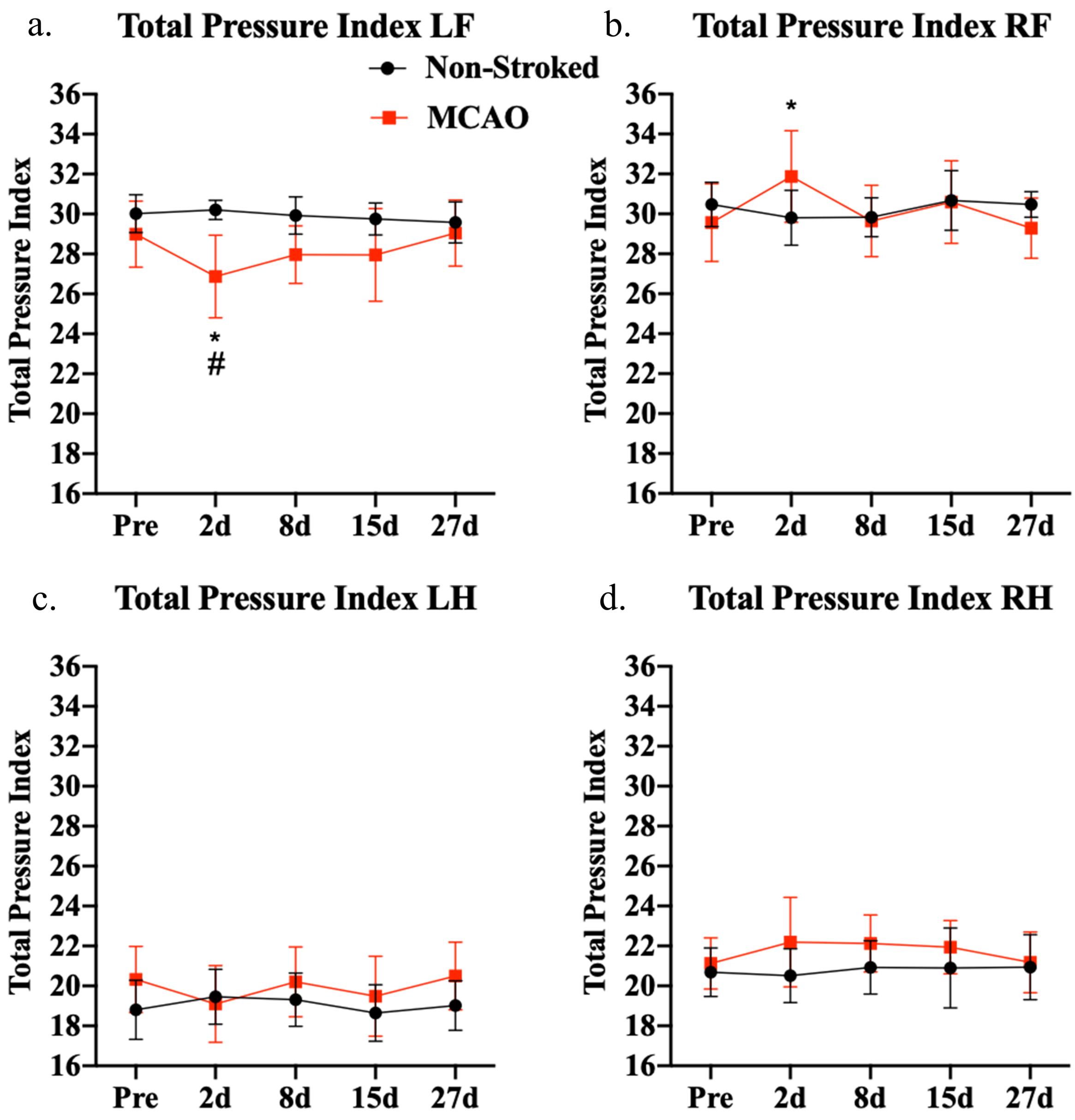

Supplementary Figure 5: MCAO induced limb weakness as measured by total pressure index. Total pressure index in the left front (LF) decreased compared to pre-MCAO and was significantly different than non-stroked controls at 2d post-MCAO (**a**). Total pressure index in the right front (RF) correspondingly increased at 2d post-MCAO compared to pre-MCAO (**b**) indicating weight and strength compensation. Hind limbs did not display any significant

observed weight distribution abnormalities (**c**, **d**). * indicated statistical difference from pre-MCAO values. # indicated statistical difference between groups.

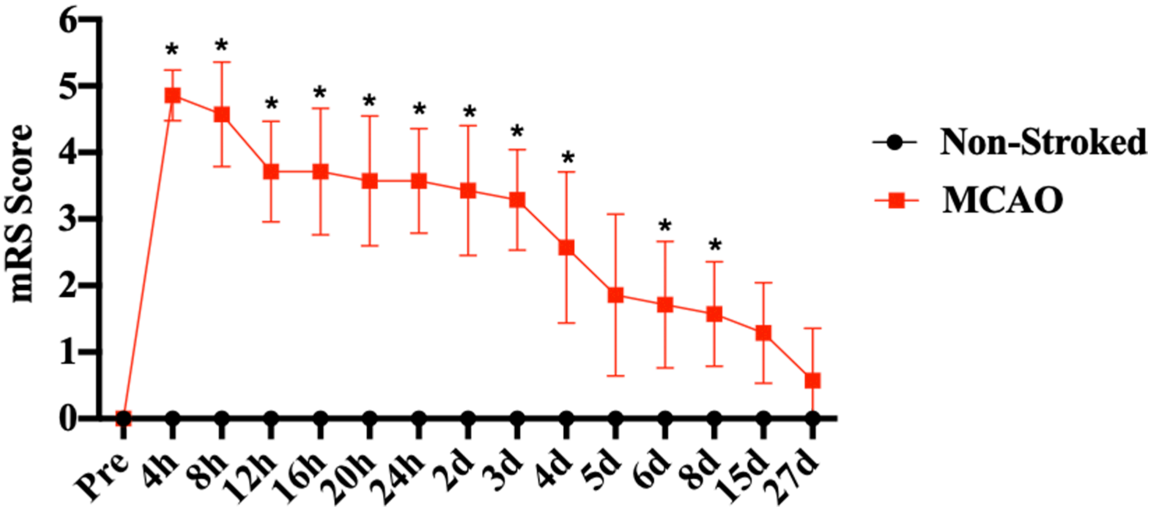

Supplementary Figure 6: Modified Rankin Scale score quantified behavioral deficits induced by stroke. Modified Rankin Scale (mRS) score is highest in the acute phase and statistically ($p<0.05$) different than pre-MCAO values through 8d post-MCAO. * indicated statistical difference from pre-MCAO values.

References

1 Spellicy, S. E. *et al.* Neural Stem Cell Extracellular Vesicles Disrupt Midline Shift Predictive Outcomes in Porcine Ischemic Stroke Model. *Transl Stroke Res*, doi:10.1007/s12975-019-00753-4 (2019).
